# Isolated splenic lymphangioma in adulthood

**DOI:** 10.1093/jscr/rjae411

**Published:** 2024-06-11

**Authors:** Mohsin Yahya Murshid, Atif Omar AlHawsah, Kadi T AlSayed, Farrukh Alim Ansari

**Affiliations:** Department of General Surgery, International Medical Center, P.O. Box 2172, Jeddah 21451, Saudi Arabia; Department of General Surgery, Hera General Hospital, Al Madinah Al Munawarah Rd, Makkah 24227, Saudi Arabia; Department of General Surgery, Hera General Hospital, Al Madinah Al Munawarah Rd, Makkah 24227, Saudi Arabia; Department of General Surgery, Hera General Hospital, Al Madinah Al Munawarah Rd, Makkah 24227, Saudi Arabia

**Keywords:** lymphangioma, splenic tumors, rare tumor, splenic cyst, splenic neoplasms

## Abstract

Isolated splenic lymphangiomas are rare benign lesions mostly seen in children are exceptionally rare in adults, often discovered incidentally due to their typically asymptomatic nature. This case report elaborates on the surgical excision of a rare splenic cystic lymphangioma in a 33-year-old woman, underscoring the diagnostic and therapeutic challenges these tumors pose. The patient’s symptoms, abdominal pain and a palpable mass led to imaging through ultrasound and CT, which revealed a cystic splenic lesion. Total splenectomy was performed, revealing a large cystic mass, with pathological examination confirming a cystic lymphangioma. This case emphasizes the necessity of considering splenic lymphangiomas in adult patients presenting with splenic lesions. It also highlights the critical role of surgical interventions for definitive diagnosis and to prevent complications such as rupture and hemorrhage, thereby emphasizing on the complexity of managing rare splenic tumors.

## Introduction

Primary neoplasms of the spleen are rare clinical entities, presenting a challenge in both diagnosis and management. These lesions can be classified according to their histological origin and further into benign or malignant types depending on their nature [1]. Isolated splenic lymphangiomas represent an extremely rare subset of splenic tumors, and are result of congenital lymphatic malformation. These tumors are mostly diagnosed in pediatric populations, with diagnosis in adult patients being exceedingly rare [2]. These tumors are generally asymptomatic and are often discovered incidentally. However, when symptomatic, they are typically attributed to the mass effect of the tumor due to increasing size [3]. Cystic masses of the spleen are still challenging to accurately diagnose preoperatively. We present a case of a 33-year-old symptomatic female patient who was diagnosed with cystic lesion of spleen and underwent total splenectomy.

## Case report

**Figure 1:**
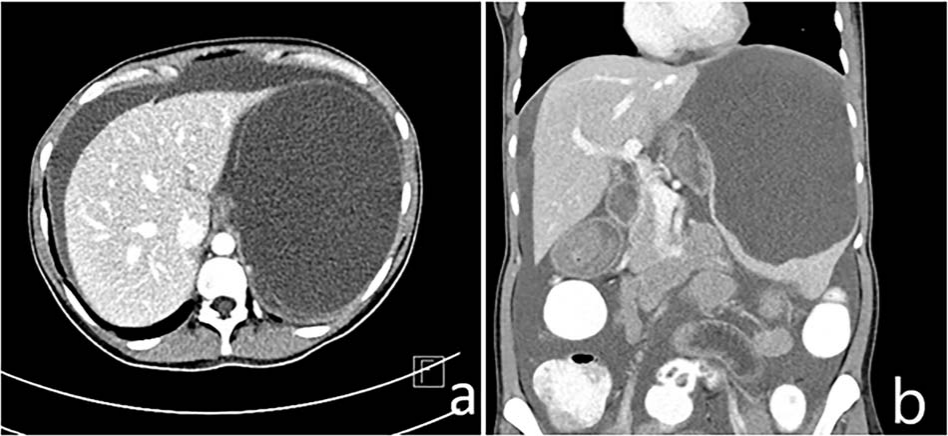
(a and b) Enlarged spleen (about 19 cm) with a large splenic cyst measuring about 15.7 × 12 × 10 cm^3^ with mass effect on the stomach displacing it to the right side with marked intraperitoneal free fluid seen (ascites).

**Figure 2:**
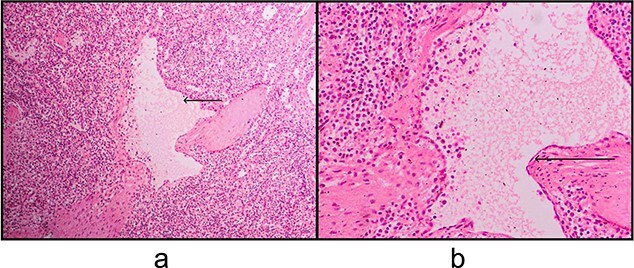
(a) Thin-walled cystic space surrounded by normal splenic parenchyma, filled with eosinophilic amorphous material ([H&E], 10x). (b) The lining of the cysts consists of attenuated endothelial cells (H&E, 40x).

A 33-year-old female patient presented to the Emergency Department with a complaint of abdominal pain for the past 4 months. The pain was mainly localized to the left upper quadrant and epigastric area and was associated with nausea, post-prandial vomiting and early satiety. On examination, a palpable mass was discovered in the left upper quadrant extending to the peri-umbilical area. There was no tenderness or guarding. There was no history of weight loss or other constitutional symptoms. Laboratory investigations were unremarkable. Other systemic examinations were also unremarkable. There was no significant family history. The patient underwent an ultrasound of the abdomen that showed a splenic hypoechoic cystic lesion with hypoechoic and hyperechoic septa. Abdominal CT imaging revealed an enlarged spleen (about 19 cm) with a large splenic cyst measuring about 15.7 × 12 × 10 cm^3^ with mass effect on the stomach, displacing it to the right side with marked intraperitoneal free fluid seen (Fig. 1). Both splenic cyst content and ascites show the same criteria on ultrasound, and the same HU density. There were no other findings detected in the abdomen. The initial differential diagnosis based on imaging included splenic cyst and neoplastic lesion. After a thorough discussion with the patient, it was decided to go forward with total splenectomy for definitive diagnosis and symptom relief. The patient was administered the required vaccines 14 days prior to the surgery as per guidelines. The patient underwent laparotomy via a midline vertical incision. The outer surface of the spleen was irregular and brownish-grey colored with multiple nodular formations. Gross sections showed cystic cavities of different sizes. The largest one measured was 11 × 5 × 9 cm^3^ with some wall calcifications. The rest of the splenic parenchyma had no remarkable changes. Histopathological examination revealed multiple cystic spaces, lined by flattened endothelial cells. The lumens were filled with proteinaceous material (Fig. 2). Immunohistochemistry was not done at our center due to unavailability. There were no intra-operative complications and post-operative recovery was satisfactory. She was discharged on the fourth post-operative day. There was mild thrombocytosis (590 × 10^9^/L) post-operatively, which returned to normal levels subsequently in the following weeks. The patient was followed up for the next 3 months with satisfactory recovery.

## Discussion

Isolated splenic lymphangiomas are extremely rare, benign primary tumors of the spleen. These are slow-growing masses formed mainly as a result of congenital malformations of the lymphatic system [4]. It accounts for 0.007% of all tumors. It is mostly seen during childhood and is rarely seen in adult patients. Splenic lymphangiomas can be part of systemic lymphangiomatosis syndromes, but no such syndrome was detected in this case. Symptoms, if present, are due to the increasing weight of the mass leading to mass effect. These tumors can be multiloculated, lobulated or cyst-like. These lesions, like hemangiomas, can be unifocal or part of systemic lymphangiomatosis syndromes that affect multiple organs. Three histologic subtypes are recognized, na,ely capillary, cavernous and cystic, with cystic type being the most common [5].

Imaging options include ultrasonography, CT scan or MRI. Ultrasound reveals numerous hypoechoic cysts with hyperechoic septa, as well as visible calcifications. A CT scan typically reveals subcapsular cysts that are finely defined, thin-walled and have low density. Peripheral mural calcifications may indicate cystic lymphangiomas; however, they are not a definitive sign [6]. On MRI, it typically presents as well-defined multilocular cystic lesions with thin septations that are uniformly hypo-intense on T1-weighted scans. However, it might also seem very intense when loaded with hemorrhagic or proteinaceous material. The lesion on T2-weighted imaging shows multiple loculated hyperintense areas that represent the dilated lymphatic channels [7]. Angiography shows distinct avascular lesions spread across the spleen, with no signs of new blood vessel formation, arteriovenous connections or blood accumulation, resulting in a unique ‘Swiss-cheese’ pattern [8, 9]. FNA-biopsy is not recommended due to the high risk of bleeding and a lack of enough tissue samples [10].

Cystic lesions of the spleen have radiological similarities and are therefore difficult to diagnose definitively on radiological findings. The gross appearance of splenic lymphangioma is varied and may include solitary nodules, multiple nodules and diffuse lymphangiomatosis [6]. Microscopically, these cysts are made up of variably sized, thin-walled vascular channels lined by a single layer of endothelial cells and filled with eosinophilic, proteinaceous material. Immunohistochemical staining includes FVIII-Rag, CD31, CD34 and D2-40 (a selective marker for lymphatic endothelium) for the identification of endothelial cell lining [11, 12].

Once the diagnosis of cystic lesions has been made, it is recommended that the patient undergo splenectomy for definitive histopathological diagnosis and to prevent possible complications like splenic rupture, hemorrhage, infection and intestinal obstruction [13]. Although total splenectomy has traditionally been the gold standard for management of these lesions, partial splenectomy is becoming an increasingly common practice, but it is associated with an increased risk of recurrence in the remnant spleen. Both open and laparoscopic approaches can be used, with the decision being based on patient characteristics, the surgeon’s expertise and the extent of spleen enlargement. The laparoscopic approach is typically the preferred option for spleens with normal to moderate enlargement. In contrast, the open method is favored for spleens that are significantly enlarged, as the laparoscopic method may be challenging and carry a higher risk of intraoperative complications in these cases [14].

The prognosis of splenic lymphangioma after complete resection is favorable. The rate of malignant transformation is very low [15]. Post-operative surveillance generally focuses on post-splenectomy complications. Recurrence is the primary complication, following a partial resection, occurring in 9.5% of patients [8]. It’s also important to adhere to vaccination guidelines for splenectomy.

## Conclusion

This case study outlines the removal of a rare splenic lymphangioma in a 33-year-old, underscoring the challenge of diagnosing such conditions in adults. Imaging aids diagnosis, but pathological examination is crucial for confirmation. Splenectomy is highlighted as essential for preventing severe complications, emphasizing the importance of accurately differentiating this condition for effective management.
